# Supplementary material for: A Temperature/pH Double-Responsive and Physical Double-Crosslinked Hydrogel Based on PLA and Histidine
Source: Gels. 2022 Sep 7;8(9):570. doi: 10.3390/gels8090570 (PMC9498346; doi:10.3390/gels8090570)
Supplement: Supplementary file 1 [file gels-08-00570-s001.zip › gels-1877244-supplementary material.pdf]

gels

MDPI

A Temperature/pH Double-Responsive and Physical Double-Crosslinked Hydrogel Based on PLA and Histidine

Qingrong Wu, Yu Fu, Wanying Yang and Shouxin Liu *

Key Laboratory of Applied Surface and Colloid Chemistry, Ministry of Education, School of Chemistry and Chemical Engineering, Shaanxi Normal University, Xi'an 710119, China

* Correspondence: liushx@snnu.edu.cn; Tel.: +86-29-8153-0781

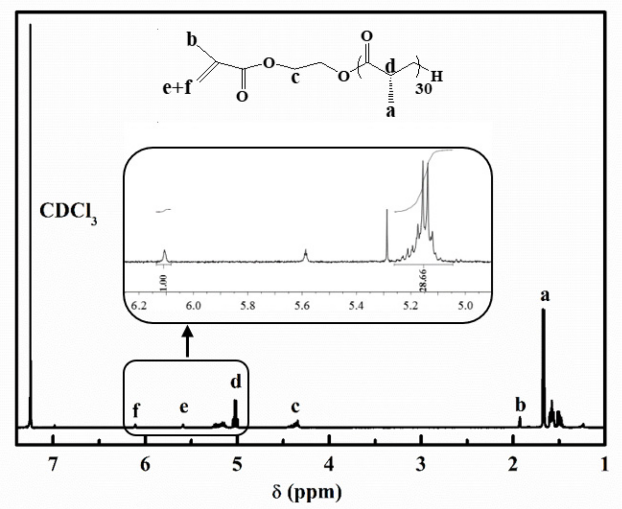

Figure S1. ^{1}H NMR spectra of macromonomer HEMA-PDLA$_{30}$.

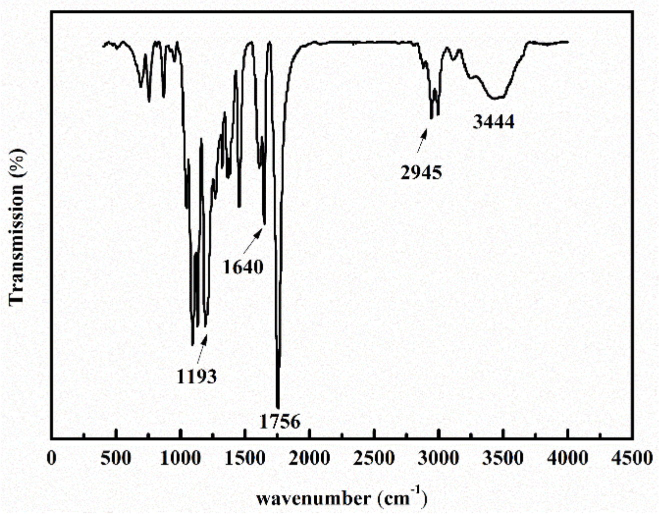

Figure S2. FTIR spectra of macromonomer HEMA-PDLA$_{30}$.

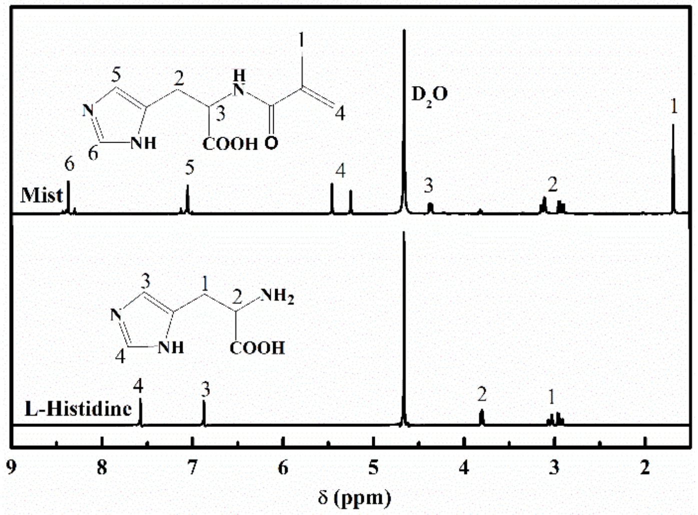

Figure S3. ^1H NMR spectra of Mist and L-Histidine monomers.

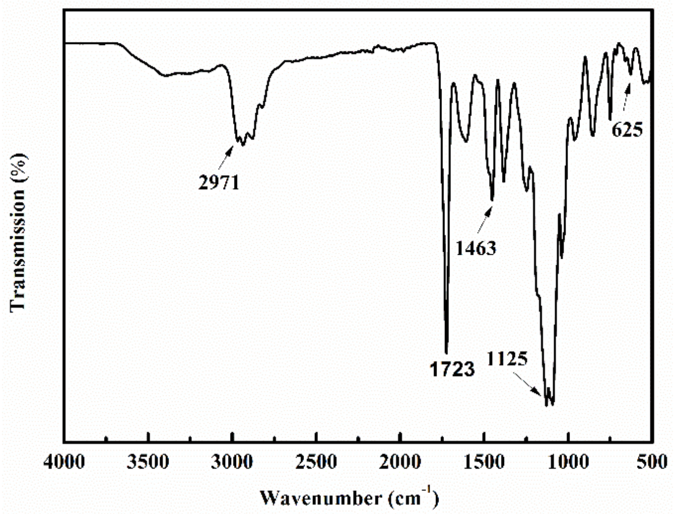

Figure S4. FTIR spectra of temperature / pH double-responsive and physical double-crosslinked hydrogel.

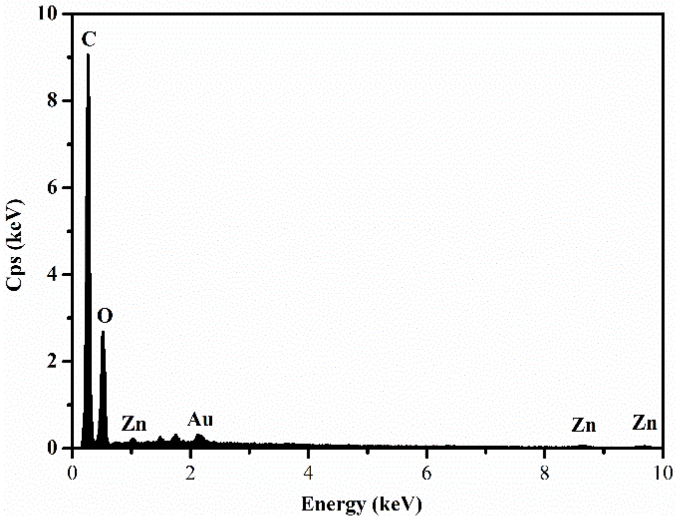

Figure S5. EDAX of double-crosslinked hydrogel.
